# Comparison of normalization approaches for gene expression studies completed with high-throughput sequencing

**DOI:** 10.1371/journal.pone.0206312

**Published:** 2018-10-31

**Authors:** Farnoosh Abbas-Aghababazadeh, Qian Li, Brooke L. Fridley

**Affiliations:** 1 Department of Biostatistics & Bioinformatics, Moffitt Cancer Center, Tampa, FL, United States of America; 2 Health Informatics Institute, University of South Florida, Tampa, FL, United States of America; Boston University, UNITED STATES

## Abstract

Normalization of RNA-Seq data has proven essential to ensure accurate inferences and replication of findings. Hence, various normalization methods have been proposed for various technical artifacts that can be present in high-throughput sequencing transcriptomic studies. In this study, we set out to compare the widely used library size normalization methods (UQ, TMM, and RLE) and across sample normalization methods (SVA, RUV, and PCA) for RNA-Seq data using publicly available data from The Cancer Genome Atlas (TCGA) cervical cancer study. Additionally, an extensive simulation study was completed to compare the performance of the across sample normalization methods in estimating technical artifacts. Lastly, we investigated the effect of reduction in degrees of freedom in the normalized data and their impact on downstream differential expression analysis results. Based on this study, the TMM and RLE library size normalization methods give similar results for CESC dataset. In addition, the simulated datasets results show that the SVA (“BE”) method outperforms the other methods (SVA “Leek”, PCA) by correctly estimating the number of latent artifacts. Moreover, ignoring the loss of degrees of freedom due to normalization results in an inflated type I error rates. We recommend adjusting not only for library size differences but also the assessment of known and unknown technical artifacts in the data, and if needed, complete across sample normalization. In addition, we suggest that one includes the known and estimated latent artifacts in the design matrix to correctly account for the loss in degrees of freedom, as opposed to completing the analysis on the post-processed normalized data.

## Introduction

Demand for revolutionary technologies to deliver fast, inexpensive and accurate information has accelerated the development of high throughput sequencing (HTS) technologies. In the last five years, massively parallel RNA sequencing (RNA-Seq) has allowed for the large scale characterization of the transcriptomic landscape of cancer. Subsequently, many methods have been developed that not only provide accurate measurements of transcript abundance [1,2], but also improved transcription start site mapping [3], gene fusion detection [4], small RNA profiling [5], investigation of alternative splicing and/or novel exons [6], and allele specific expression [7,8]. However, some issues have been reported in the completion of large-scale transcriptomic studies, such as technical variation, sequencing bias and mapping bias [9,10]. Hence, experimental design is critical for the completion of accurate and non-biased studies [11].

In completing RNA-Seq studies, variability in measurement can be attributed to both the biological and technical factors. Sources of technical variation include: differences in library preparation across samples [12], sequencing error [13], mapping and annotation bias [13], sequencing composition and similarity [14,15], gene length [12,15], and sequencing depth [12]. Such unwanted noises can significantly reduce the accuracy of statistical inferences from RNA-Seq data [16–18] and also prevent researchers from properly modeling biological variation and group-specific changes in gene expression [19]. In addition to correcting for known differences outlined above, one also needs to assess for unknown or “latent” effects, and if needed, complete normalization to adjust for these known and unknown artifacts in the data. Hence, thoughtful consideration is needed in determining how to normalize the RNA-Seq data. Similar to analysis of microarray based gene expression data, the results of differential gene expression analyses can be noticeably influenced by the choice of normalization method to remove the technical artifacts [12,20,21]. Arguably, choice of normalization approach can have a larger impact on the downstream analysis results than the choice of method used for completing differential expression [12].

In recent years, various normalization methods were proposed to correct for technical biases. Type of technical biases can be divided into three general varieties: (1) normalization for gene length, (2) normalization for library size (i.e, sequencing depth), and (3) normalization for known or unknown technical artifacts across samples. Such normalization methods differ in the type of biases adjustment and statistical assumptions. **Table 1** presents the most recent proposed normalization methods for these three types of normalization approaches. In the last few years, a number of comprehensive studies have done to compare different normalization approaches to treat RNA-Seq data and their impact on the downstream analysis, where several publications have focused on the normalization methods that correct for differences in library size and/or gene length [12,22–24]. In contrast, a number of studies have been completed to compare methods for known or unknown technical artifacts across samples than simply in sequencing depths [17,18,25–27]. However, none of the previous studies did the comprehensive comparison of the library size and across sample normalization methods, where the impact of loss of degrees of freedom due to normalization for downstream differential expression analysis was taken into account.

**Table 1:** Summary of current normalization methods to correct the technical biases for RNA-Seq data.

Technical Bias	Normalization Method	Reference
**Library Size**	Total count (TC)	Dillies M. A. et al. [22]
	Median	Dillies M. A. et al. [22]
	UQ	Bullard J. H. et al. [12]
	TMM	Robinson and Oshlack [21]
	RLE	Anders and Huber [34]
	Quantile (Q)	Smyth G. K. [49]
**Across Sample**	PCA	Price A. et al. [40]
	RUV	Risso D. et al. [17]
	SVA	Leek and Storey [18]
**Gene Length**	TPM	Mortazavi A. et al. [15]
	FPKM / RPKM	Mortazavi A. et al. [15]

Therefore, the goals of this study are the following: 1) provide a review of existing methods for normalization of RNA-Seq data; 2) to assess the various methods for library size normalization using a large expression study in data; 3) to assess the various methods for determining latent or unknown artifacts in the data using both real and simulated data; and 4) to assess the impact of not accounting for the loss of degrees of freedom due to normalization and the impact on the type I error rate for differential expression analysis. The methods are compared using data from The Cancer Genome Atlas (TCGA) cervical study (CESC) [28]. Moreover, an extensive simulation study was completed to allow for further investigation into the performance of the across sample normalization methods in estimating the latent artifacts along with the impact on the operating characteristics for differential expression testing due to the reduction in the degrees of freedom due to normalization.

## Materials and methods

**Fig 1:**
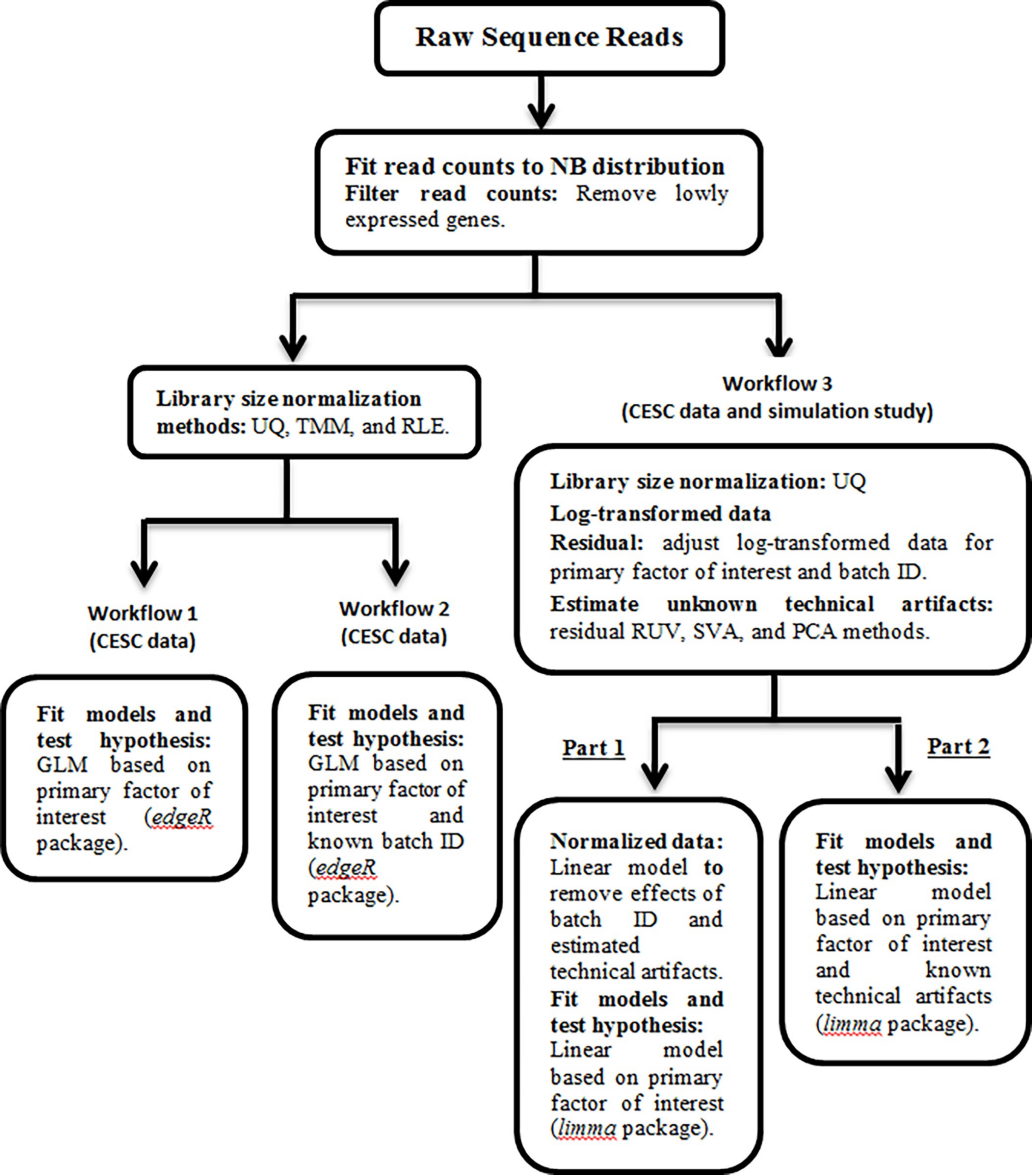
Flow chart showing analysis plan for comparing the effects of different types of normalization methods on differential expression analysis. Using CESC dataset to compare the effect of library size normalization methods on the differential expression analysis using workflow 1 (design matrix contains primary factor of interest) and workflow 2 (design matrix contains batch ID along with the primary factor of interest). Workflow 3 compares the different methods to estimate the latent artifacts followed by across sample normalization for these unknown factors, and considers the impact of not accounting for loss of degrees of freedom due to normalization for differential expression analysis using both CESC dataset and simulated data. For both simulated and CESC data, we considered two approaches to determine DE genes (i.e., workflow 3): (Part 1) analysis based on post-normalization, and (Part 2) normalization for batch effects is completed through the design matrix that also includes the primary variable of interest. Note that under the workflow 3, the UQ normalization method was not considered for the simulated data, and also there is not any known technical artifact (e.g., batch ID).

In this section, we describe the normalization methods used in our study as well as the specific criteria used in our comparison. We also discuss the TCGA study and the simulation study used to assess the methods. We examined the effects of different normalization methods on the differential expression analysis using three analysis workflows (**Fig 1**). Here workflows 1 and 2 compare the effect of library size normalization methods on the differential expression analysis, while workflow 3 compares different methods to estimate the latent artifacts followed by across sample normalization for these unknown factors, and also considers the impact of not accounting for loss of degrees of freedom due to normalization for differential expression analysis. Note workflows 1–3 are used by TCGA cervical study, and for the simulation study, only workflow 3 is considered.

### Gene length normalization

A technical bias not observed with microarray data but observed in the completion of RNA-Seq studies is the impact of gene length on estimation of gene abundances. In particular, larger genes will have inevitably higher read counts compared to smaller genes due to the difference in their gene lengths or sizes [15]. One often method to correct for this bias is the use of RPKM/FPKM (reads/fragments per kilo-base per million mapped reads) [15,29,30]. Another approach to adjust for gene length is the TPM (transcripts per million) method, which takes into account both the gene length and the sequencing read length corrections; however, it can still suffer some biases such as sequencing depth and latent technical artifacts [10,24]. ERPKM is an improvement of RPKM that replaces gene length with an effective read length (i.e., gene length–read length + 1) [23]. These approaches re-scale gene counts to correct the differences in gene length, as illustrated in **S1A Fig**.

All these methods rely on normalizing approaches based on the total or effective counts, and tend to perform poorly when samples have heterogeneous transcript distributions [12,31]. Scaling counts by gene length can give biased estimates of differential expression and the clearly positive association between gene length and counts is not entirely removed by applying the gene length normalization [10,12,30,31]. However, TPM and RPKM/FPKM values are appropriate to use if the goal is to compare the expression levels between genes (i.e., across gene comparison), the differential expression analysis needs that the expression levels should be compared across samples [15,24].

### Library size normalization

One source of variation between samples is the difference in library size, where library size is the total number of reads generated for a given sample. Difference in library size can be due to many factors, including differences in multiplexing of samples (allocation of samples to lanes in a flow cell) or global differences in gene expression levels (**S1B Fig**). The goal of library size normalization is to make the library sizes comparable by scaling raw read counts in each sample by a single sample-specific factor reflecting its library size. There are three commonly used methods: Upper Quartile (UQ), Trimmed Mean of M-values (TMM), and Relative Log Expression (RLE).

- **Upper Quartile (UQ)**: Under this normalization method, after removing genes having zero read counts for all samples, the remaining gene counts are divided by the upper quartile of counts different from zero in the computation of the normalization factors associated with their sample and multiplied by the mean upper quartile across all samples of the dataset [12]. This normalization method is implemented in the *EDASeq* and *edgeR* Bioconductor packages [32,33].
- **Trimmed Mean of M-values (TMM)**: This normalization method is based on the hypothesis that most genes are not differentially expressed (DE). For each sample, the TMM factor is computed while one sample is considered as a reference sample and the others as test samples. For each test sample, after exclusion of the most expressed genes and the genes with the largest log ratios, TMM is computed as the weighted mean of log ratios between this test and the reference. Because of the low DE hypothesis, the TMM should be close to 1. If it is not, its value provides an estimate of the correction factor that must be applied to the library sizes [21]. This normalization method is implemented in the *edgeR* Bioconductor package as the default normalization method [33].
- **Relative Log Expression (RLE)**: Similar to TMM, this normalization method is based on the hypothesis that the most genes are not DE. For a given sample, the RLE scaling factor is calculated as the median of the ratio, for each gene, of its read counts over its geometric mean across all samples. By assuming most genes are not DE, the median of the ratio for a given sample is used as a correction factor to all read counts to fulfill this hypothesis [34]. This normalization method is included in the *DESeq* and *DESeq2* Bioconductor packages [34,35].

### Across sample normalization

As the library size normalization methods mostly correct for sequencing depth and fail to adjust for other technical variations, across sample normalization methods have been proposed to correct for other technical artifacts to improve data quality and ability to detect biologically relevant genes. However, such technical sources of variation become problematic to deal with when they are correlated or confounded with a primary biological factor of interest; therefore good experimental design is essential when completing HTS studies. Thus, in completing normalization for technical artifacts we often assume that the primary factor of interest is independent of all artifacts [16,17,25]. Additionally, many possible sources of technical variations are not recorded or unknown to the researcher. Therefore, in addition to normalizing for known technical artifacts, assessment and adjustment for potential unknown or latent variables is also warranted [18].

### Known technical artifact

In contrast to the more complex modeling methods is the approach involving the direct adjustment for known technical artifacts within the appropriate statistical model (e.g., linear regression models). As an example, a model with the primary biological factors of interest can be included in the design matrix along with the known technical artifacts, which may improve the precision of the comparisons of interest [16,36]. In addition, some proposed normalization methods are not robust to outliers in small sample sizes, where a flexible empirical Bayes method, referred to as *ComBat*, was proposed to provide more robust batch effect adjustments with small sample sizes [26,27]. However, the *ComBat* approach places less emphasis on the biological model and mostly reducing global variation even without specifying a biological model. In addition, the *ComBat* approach uses an empirical Bayes method to avoid over-correcting the known technical artifacts, which is critical for use with small batches (or sample size).

### Unknown technical artifact

Recently, normalization methods have been developed to assess and remove unknown technical variations by estimating the latent factors to capture these sources of variation. Several of these methods rely on singular value decomposition (SVD) or some other factor analysis techniques to determine the unwanted variation directly from the data. One issue with applying these approaches is the difficulty in distinguishing the unwanted technical variation from the biological factors of interest. Hence, remove unwanted variation (RUV) method was proposed to adjust for unknown technical variations by performing factor analysis on negative control genes (i.e., genes known not to be related to the primary factor of interest) [17,37]. Therefore, variations in the expression levels of these genes can be assumed to be unwanted variations. Housekeeping genes [38] or spike-in controls [39] are the examples of negative controls. However, RUV method does not need the negative control genes or samples [17]. Other commonly used methods to address this problem in identifying the unknown technical variations are the surrogate variable analysis (SVA) [18] and principal component analysis (PCA) [40]. It should be noted that in the case of RUV, SVA and PCA methods, it is possible that some of the estimated latent factors are not technical artifacts but rather represent true biology presented in the data. Thus, it is important to adjust for any known biological factors of interest and known technical artifacts prior to estimation of latent factors. The correct usage of these methods in estimating the latent technical artifacts has the potential to increase statistical power in downstream differential expression analysis, while note that increasing the number of estimated batch effects also can reduce power due to the additional bias of degrees of freedom [27,41].

After estimating the latent factors using RUV, SVA and PCA approaches, an appropriate statistical approach (e.g., linear model or *ComBat*) is used to obtain the normalized data.

- **Remove Unwanted Variation (RUV)**: Under this approach, the factors of unknown technical variations are estimated and removed by performing the factor analysis on suitable sets of negative control genes or samples by keeping the primary factor of interest. Therefore, RUV [17] method is divided into three sub-methods: RUVg, RUVs, and RUVr. The RUVg and RUVs are used when negative control genes and negative control samples (i.e., samples whose read counts are not influenced by the primary factor of interest) exist. However, RUVr (i.e., residual RUV) does not require the existence of negative control genes or samples. SVD is then computed on the residual matrix to estimate the factors of unknown technical variations. The number of factors of unwanted variation, k, should be guided by considerations that include samples sizes, extent of technical effects captured by the first k factors, and extent of differential expression [17,25].
- **Surrogate Variable Analysis (SVA)**: In this approach, the unknown technical variations or “surrogate variables” (SV’s) are estimated by applying SVD on the computed residual matrix and selecting significant eigenvectors [18,25,42]. The first step in SVA is to determine the number of SVs using one of the two methods, “BE” or “Leek” as noted in [18,26,43]. The “BE” method is based on a permutation procedure originally proposed by Buja and Eyuboglu [43], while the “Leek” method provides an interface to the asymptotic approach proposed by Leek [42], where under the specific assumptions, the right singular vectors are asymptotically consistent for latent artifacts as the number of features grows large. Once the number of SVs is calculated, then using the two-step algorithm following Leek and Storey [18] to estimate unknown technical artifacts.
- **Principal Component Analysis (PCA):** This approach is completed by applying SVD to the scaled residual matrix to estimate the factors of unknown technical variations [44]. One can determine the number of PCs to include in the model by multiple methods, including: PCs that explain a given percent of the variation; PCs that are associated with the biological factors of interest (i.e., confounders); top PCs regardless of association with the primary factors of interest; application of the Tracy-Widom test for determining eigenvalues significantly different from zero (noting that the assumption of independence is not valid) [45–47]; or determine the PCs to include based on a permutation testing approach similar to that implemented in the SVA method.

### Issues of loss of degrees of freedom

For practical purposes it is more convenient to perform downstream analyses on the batch adjusted or normalized data without further consideration of technical artifacts effects. However, adjustment for technical artifacts reduces the effective degrees of freedom in the dataset and thus changes the null distribution of the test statistics. Not accounting for this change in the degrees of freedom due to normalization for batch effects may lead to increase the false positive rates, especially when the primary factors of interest are not equally represented in all batches or batch effects act as a confounder [41]. It should be noted that whatever our normalization approach is, one is in essence reducing the degrees of freedom in the data which in turn should lower the statistical power of the test. For example, let’s assume there are two studies (Study A and Study B) with the same sample size. Let’s also assume that Study A implemented good experimental design and did not need to normalize for any known or unknown technical artifacts, while Study B did not implement adequate experimental design and thus needed extensive across sample normalization. In this situation, Study A will have more power to detect a true biological effect compared to Study B. However, the loss of degrees of freedom associated with Study B due to normalization is often overlooked in the implementation of analysis approach involving across sample normalization followed by association analysis of gene expression levels with the biological factor of interest. In the following sections, we assess the extent of the impact of the loss of degrees of freedom on the type I error rate on association testing via a simulation study.

### Comparison of methods

#### TCGA cervical study

To compare the normalization methods and their impact on the differential expression analysis, publically available data from the TCGA cervical study (CESC) was used [28]. Level 3 RNA-Seq data (summarized gene expression levels) and clinical patient data were downloaded via Genomic Data Commons (GDC) (https://gdc.cancer.gov/) (July 2017). The large-scale study the size of the TCGA unavoidably generated technical artifacts. These factors (e.g., tissue source site, plate ID, sequence center) were tracked for each sample with this information contained within the original TCGA ID. These known factors can also be downloaded from *MBatch*, a web-based analysis tool for normalization of TCGA data developed by MD Anderson. (https://bioinformatics.mdanderson.org/tcgabatcheffects).

For the CESC study, gene expression data was measured on 60,433 genes and 178 cervical tissue samples (144 squamous cell carcinomas, 31 adenocarcinomas and 3 adenosquamous cancers). The integrative clustering analysis reported in the main CESC TCGA paper used mRNA, DNA methylation, miRNA and copy number variation data identified two squamous-carcinoma-enriched groups and one adenocarcinomas-enriched group [28]. The two squamous-carcinoma groups differ largely based on gene expression levels where one squamous cluster had high expression of keratin gene family members (keratin-high) and the other squamous cluster had low expression of keratin genes (keratin-low). Hence, for comparison of the normalization methods and impact on the differential expression analysis results, we restricted our analysis to the squamous cell carcinomas and set out to determine DE genes between the keratin-high (N = 47) and keratin-low (N = 86) tumors groups. After filtering non-expressed or low-expressed genes based on counts per million (CPM), 20,884 genes with CPM values above 1 in at least 3 libraries remain.

#### Simulation study

An extensive simulation study was completed to compare the performance of SVA (“BE” and “Leek”) and PCA (based on different percent of variation) methods to identify the number of latent factors (i.e., SVs), and determine SVs, where the residual RUV method was also included. Then, the performance of different across latent factor identification methods were followed by normalization compared using Euclidean distance. The simulation study also investigated the impact of not accounting for the loss of degrees of freedom due to normalization on testing (i.e., impact on the type I error rate). The empirical type I error rate was computed for each “null” gene in which the proportion of the simulated datasets (out of 1,000 simulations) with differential gene expression p-value less than 0.05. Then, for the set of simulated null genes, the average type I error rate was computed by averaging the individual “null” gene type I error rates. In the simulation of the data, we considered two main scenarios: (I) only the batch effect(s) simulated (no primary biological factor); and (II) batch effects plus the effect of a biological variable of interest, where batch and biological effect were uncorrelated. The technical artifact was simulated to represent different mechanisms: discrete number of batches or runs of the samples (e.g., two groups) or a trend effect due to time of run with a continuous effect. The primary biological effect simulated was a binary factor, such as a treatment group and a control group. Note that in the simulation study, the genes with p-values less than 0.05 are considered to be DE.

We have *g* = 1,…,*G* genes, *n* = 1,…,*N* samples, *k* = 1,2 biological groups, and *l* = 1,…,*L* batches. Let *x*_*gnkl*_ be the count for gene *g* in biological group *k*, sample *n*, and batch *l*, with a Negative Binomial distribution: *x*_*gnkl*_~*NegBin*(*μ*_*gnkl*_,*φ*_*g*_). The parameters *μ*_*gkl*_ and *φ*_*g*_ are the mean and dispersion, respectively. Under each scenario (I or II), we changed the sample size (N = 20, 50, 100, 200), the percentage of genes were affected by the batch effect(s) (5%, 10%, 15%), and the percentage of DE genes (0%, 3%, 5%, 10%, 15%). For each scenario, 1,000 datasets were generated, where for each dataset we simulated expression levels for G = 1,000 genes. The baseline parameters $\mu_{g}^{*}$ and $\varphi_{g}^{*}$ (no batch or biological effects, “null” hypothesis) were estimated using the maximum likelihood estimation (MLE) for the keratin-high samples from the CESC data. The different “non-null” simulated datasets were generated as follows.

Under the scenario I, we considered the binary batch (i.e., sequencing in different labs), continuous batch (e.g., time of day), and both binary and continuous batches. While under the scenario II, we took into account the effect of primary biological factor along with the batch effects (both binary and continuous). Then, the non-null genes (i.e., DE genes) were affected by the batch effect(s) or/and primary biological factor were generated using a mean shift $\mu_{g}^{*}\;e^{\sum_{j=1}^{J}\omega_{gj}\gamma_{nj}}$ and dispersion $\varphi_{g}^{*}$, where ***ω***_*j*_ = (*ω*_1*j*_,*ω*_2*j*_,…,*ω*_*Gj*_) and ***γ***_*j*_ = (*γ*_1*j*_,*γ*_2*j*_,…,*γ*_*Nj*_) represent the *j*^*th*^ effect of batch or primary biological variable, and J represents the total number of variables as batch and primary biological in the study (see **S1 Table**).

For the scenario I, to generate only the binary batch variable (i.e., *l* = 1,2),***γ***_1_ was generated from the Bernouli distribution and ***ω***_1_ was generated from the Normal distribution (*μ* = 0,*σ* = 2). While for the continuous batch variable (i.e., *l* = 1,…,*N*),***γ***_1_ was generated from the standard Uniform distribution, *U*(0,1), and ***ω***_1_ were generated from the Normal distribution (*μ* = 0,*σ* = 6). If we consider both binary and continuous variables (J = 2), then ***γ***_*j*_ and ***ω***_*j*_ for j = 1,2 were generated as explained before. Lastly, for the scenario II, to take into account the effect of biological factor along with the batch effects (both binary and continuous), then ***γ***_3_ and ***ω***_3_ were generated from the Bernoulli distribution and the Normal distribution (*μ* = 0,*σ* = 2), respectively. The reason of changing the values of *σ* is that to provide the moderate effects for batch and primary biological variable under each scenario. The code to generate the simulated data is available at **S1 File.**

## Results

### TCGA cervical study

The filtered raw counts data from the CESC TCGA study were used to compare the effect of different normalization methods for library size and technical artifacts using three analysis workflows (**Fig 1**). To compare the library size normalization methods in terms of their impact on the differential expression analysis results, we use the *edgeR* Bioconductor package, that implements a Negative Binomial distribution where the empirical Bayes method is used to estimate dispersion parameters [33]. Under the workflow 1, we consider the library size normalization methods (UQ, TMM, and RLE) on filtered raw counts data, and fit generalized linear model (GLM) to detect DE genes, while the design matrix contains only the primary factor of interest (i.e., keratin-low and keratin-high groups). For the CESC dataset, the downloaded known technical artifacts included: tissue source site (31 levels); plate ID (21 levels); batch ID (14 levels); and ship date (20 levels). Therefore, under workflow 2, we update the design matrix used in workflow 1 by adding batch ID. However, we noted that the Fisher’s exact test between the categorical batch ID variable was not associated with the primary factor of interest (i.e., p = 0.31). This lack of association between batch ID and the primary factor of interest could be a results of low power to detect an association due to the large number of levels of batch ID (14 levels) and sparsity of the data table (i.e., 15 cell counts < 5). Lastly, workflow 3 captures the effect of unknown technical artifacts using different methods to estimate the latent artifacts in data (SVA, residual RUV, and PCA) followed by across sample normalization for these unknown factors (after applying the UQ library size normalization method). As we mentioned before, it is important to keep in mind that TMM and RLE methods rely on strong assumptions that most genes are not DE [21,34]. In practice, such assumptions are not met in some RNA-Seq data, and even checking these assumptions would be extremely difficult. Lastly, we assessed the impact of not accounting for the loss of degrees of freedom on the differential expression analysis results using the Bioconductor packages *limma* by fitting the model on: (1) the normalized data (i.e., adjusted log-transformed UQ normalized data by removing both known batch ID and estimated latent artifacts) where the design matrix contains only the primary factor of interest, and (2) the log-transformed UQ normalized data where the known batch ID and estimated batch effects are included in the design matrix along with primary factor of interest. Note that the SVA method uses the log-transformed data to estimate the SVs, then, the log-transformed data is used instead of counts data to compare the performance of different methods to estimate the latent artifacts. To compare the normalization methods in terms of their impact on the differential expression analysis results, we considered the number of DE genes and number of common DE genes found among the methods. The raw p-values were adjusted for multiple comparisons by the Benjamini-Hochberg (BH) procedure [48], which controls the false discovery rate (FDR). Then, the genes with adjusted p-values less than threshold 0.05 are considered to be DE. The results from these workflows are presented in the following sections.

#### Library size normalization

**Fig 2:**
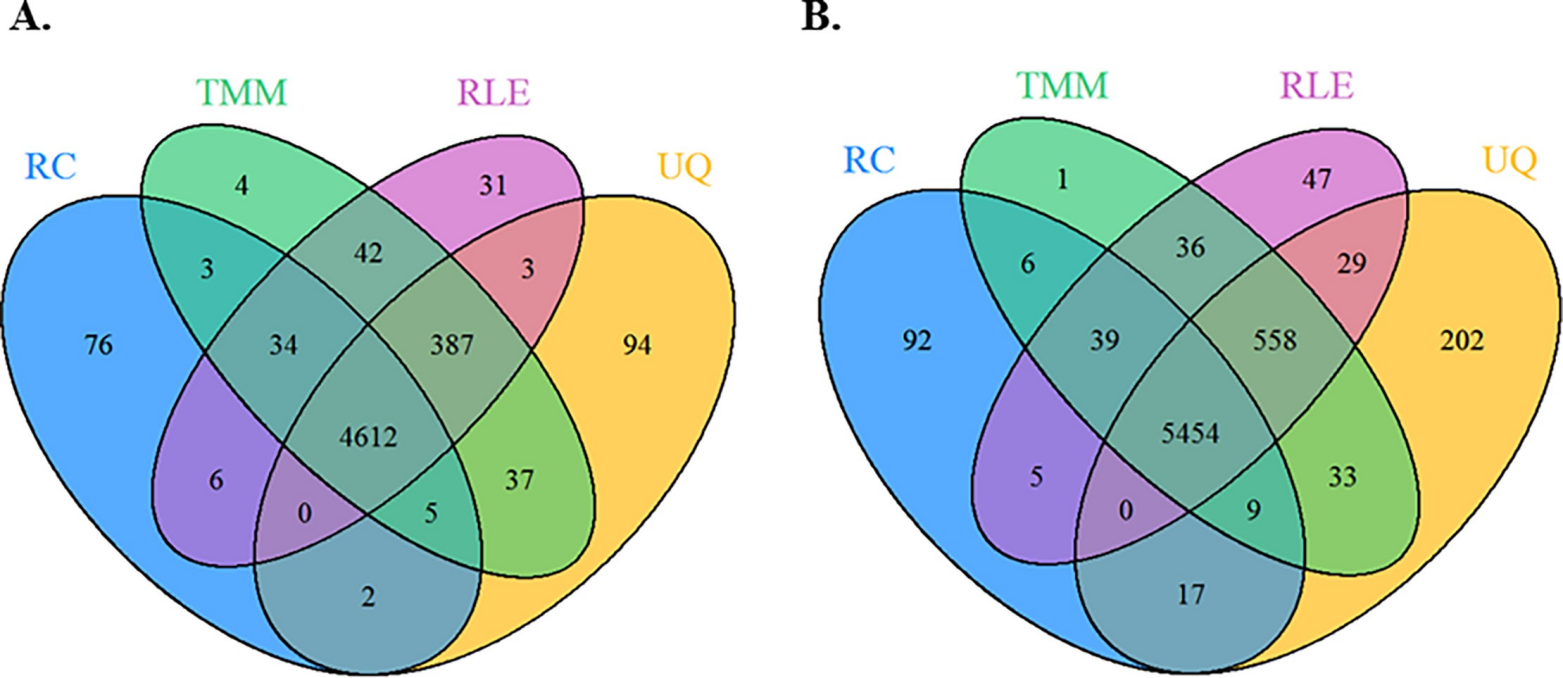
Comparison of genes with adjusted p < 0.05 from analyses of the CESC study involving the raw counts (RC) or the library size adjusted counts using the UQ, TMM or RLE methods. Design matrix contains: (**A**) workflow 2: known technical artifact (batch ID) along with the primary factor of interest (i.e., keratin-low and keratin-high groups), and (**B**) workflow 1: only the primary factor of interest.

We compared the performance of three library size normalization methods, UQ, TMM, and RLE. Analyses with and without inclusion of the known technical artifact (batch ID) (workflow 2 and 1) showed quite similar results for the different library size normalization methods (**Fig 2**), with 4,612 and 5,454 of the DE genes (adjusted p < 0.05) overlapping between methods. Most of the differences between these two workflows were due to the inclusion of batch ID in the model. Library size normalization using the UQ found the DE genes 5,140 (**Fig 2A**) and 6,302 (**Fig 2B**) from the analyses with and without adjusting for batch ID, respectively; while, the TMM (5,124) and RLE (5,115) had the fewest DE genes detected, regardless of adjustment for batch ID. However, the number of DE genes did not vary much between any of the methods. Lastly, we observed that regardless of library size normalization method, the adjustment for known technical artifact resulted in a decrease of approximately 20% detected DE genes (**Fig 2A**).

#### Across sample normalization

For comparison of methods for estimation of latent factors, we first estimate the number of latent factors found from SVA using the “Leek” method on the residual expression matrix (i.e., removing the effects of batch ID and the primary factor of interest) after using UQ library size normalization method. This approach determined three significant latent factors, while under the “BE” approach estimated 24 SVs. Thus, for the remaining methods (residual RUV and PCA) which do not determine the number of significant SVs, we estimated the top three latent factors to correspond with the number of SVs determined from the SVA (“Leek”) method. For the PCA method, the top three PCs each explained 10%, 6.2% and 5% of the proportion of total variation. A comparison between the top estimated latent factors between the methods showed that the SVA (“Leek”) and the residual RUV estimates were highly correlated (r = -0.88 and p < 2.2e-16). In addition, the top estimated latent factor based on the PCA were significantly correlated with the top estimated latent factor based on the residual RUV (r = 0.42 and p = 4e-07), and the top SVA estimated latent factor (r = -0.23, p = 8e-03).

**Fig 3:**
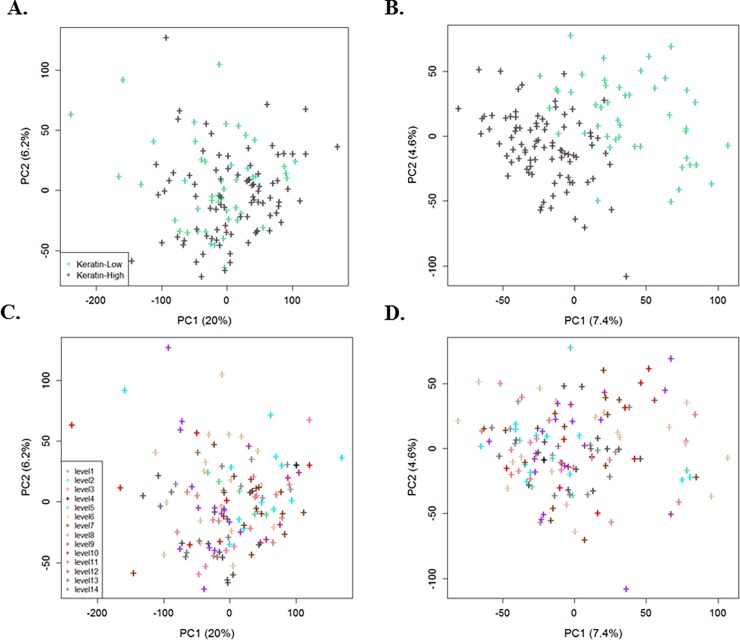
Plots of the first two PCs with the proportion of total variation for the CESC data to cluster primary factor of interest (keratin-low and keratin-high groups): (**A**, **C**) raw counts data, and (**B**, **D**) after adjusting the library size (UQ) and removing the effect of batch ID and estimated SVs by use of PCA through normalization. Each point is colored based on either the two keratin groups (biological factor) (**A**, **B**) or the14 levels of known batch ID (**C**, **D**).

**Fig 3** represents the 2D plot of first two PC’s for raw counts compared with the UQ library size normalization followed by use of PCA to demonstrate the effect of across sample normalization, where the data was normalized using a model that adjusted for known batch ID and estimated technical artifacts. **Fig 3A** and **3C** present the raw counts where the batch ID and library size had yet to be adjusted. In addition, **Fig 3C** shows the large effect of batch ID with high proportion of variation for top PC that leads to consider the workflow 2. After adjusting the library size and removing the effect of batch ID and estimated SVs (using PCA method) through normalization, we observe a clearer separation of samples with regards to the biological factor (**Fig 3B**) with less clustering of samples based on batch ID (**Fig 3D**). We found similar results for the residual RUV and SVA (“Leek”) methods (**S2 Fig**).

**Fig 4:**
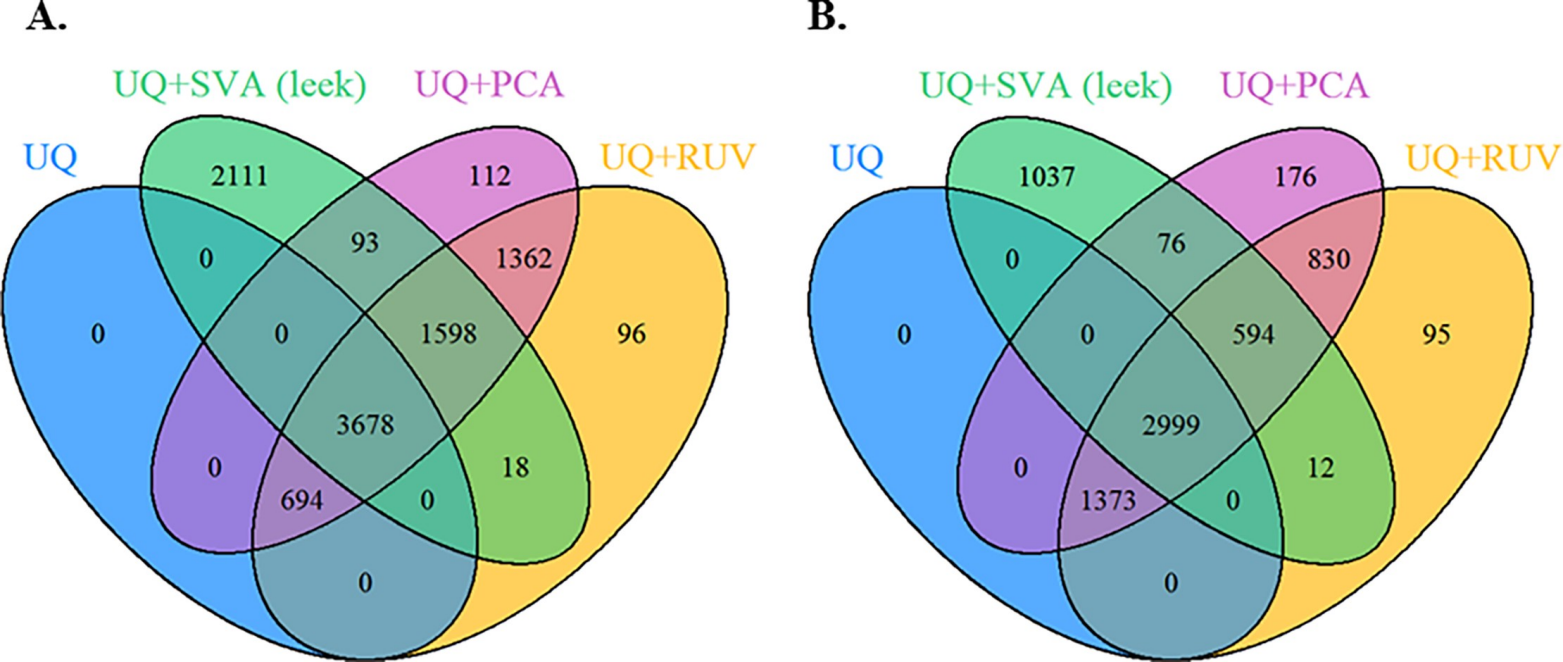
Assess the impact of correctly accounting for loss of degrees of freedom due to normalization using workflow 3. DE genes found (adjusted p < 0.05) under the UQ library size normalization method and the methods for determining the latent artifacts (SVA “Leek”, residual RUV, and PCA) for CESC data: (**A**) the normalized data (i.e., adjusted for known batch ID and estimated latent artifacts), where design matrix contains only the primary factor of interest (i.e., keratin-low and keratin-high groups), (**B**) log-transformed UQ normalized data, where the design matrix contains the estimated technical artifacts and the known technical artifact (batch ID) along with the primary factor of interest.

Next, the effect of these methods on the differential expression analysis was assessed under the workflow 3, where the goal is to assess the impact of correctly accounting for loss of degrees of freedom due to normalization. **Fig 4A** presents the overlap of DE genes (adjusted p < 0.05) between the various methods to determine unknown technical artifacts, where design matrix contains only the primary factor of interest, while in **Fig 4B** presents the results following the log-transformed UQ normalized data, where the design matrix contains the estimated latent technical artifacts and the batch ID along with the primary factor of interest. **Fig 4A and 4B** show a large overlap in the detected genes when normalization is completed for library size, known batch effects and unknown latent factors (5,276 genes), compared to 3,593 genes when using the log-transformed UQ normalized data. As the Venn diagrams illustrate (**S3 Fig**), there was a large overlap in the DE genes between using the normalized data and log-transformed UQ normalized data, where more DE genes were detected using the normalized data based on the SVA (“Leek”) (37%), while the PCA and the residual RUV methods show less percentage around 20%. The residual RUV and the PCA methods found the most DE genes 7,332 (**Fig 4A**) and 5,796 (**Fig 4B**), respectively, while SVA (“Leek”) detected 2,111 (**Fig 4A**) and 1,037 (**Fig 4B**) genes not detected by any of the other three analyses. Lastly, the residual RUV and PCA analyses produced more similar DE results, compare to SVA (“Leek”).

### Simulation study

**Fig 5:**
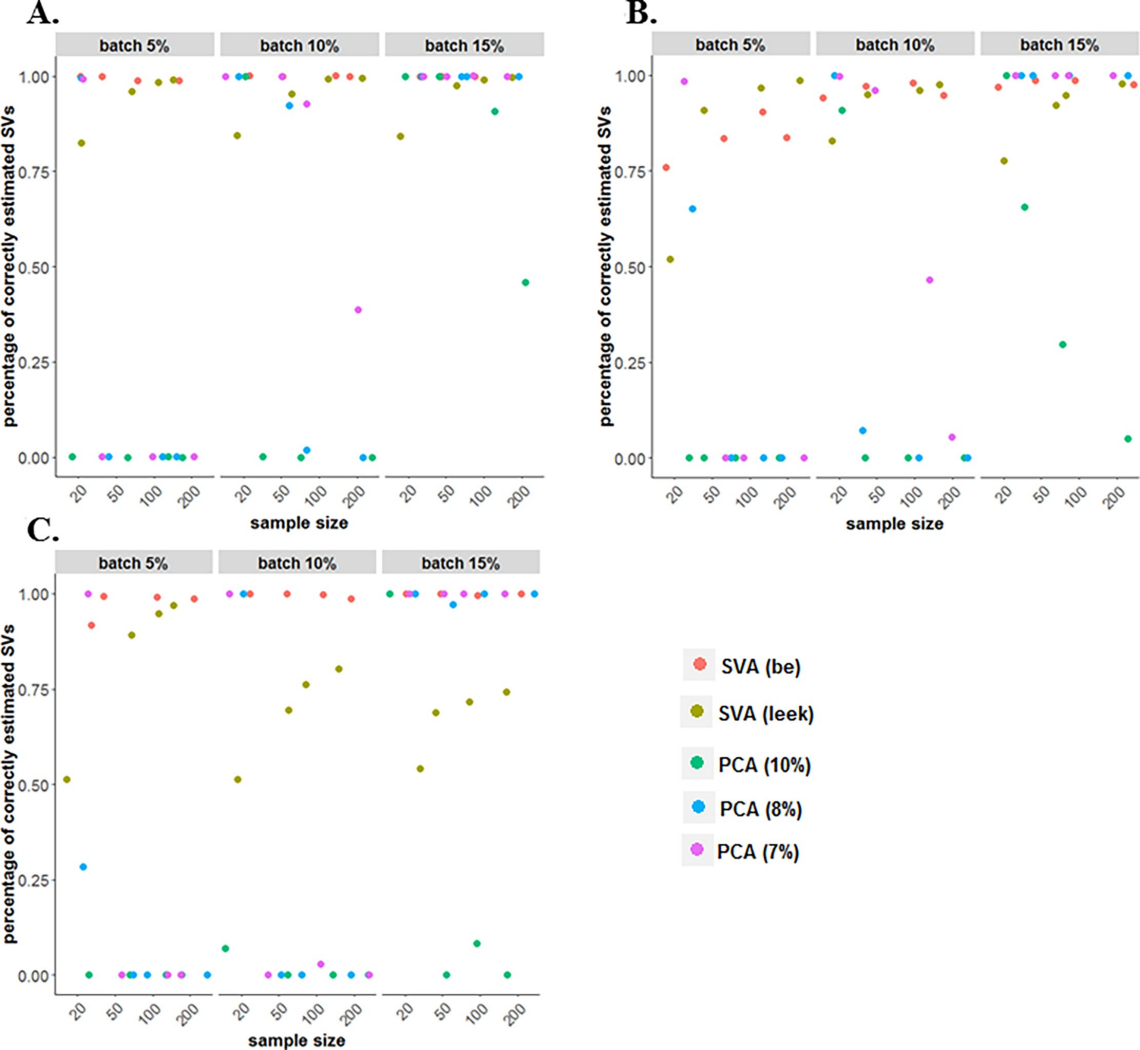
Percentage of simulated datasets with the correct number of SVs detected, were significant SVs were determined using SVA (“BE” and “Leek”) and PCA (7%, 8% and 10% percent of variation). Simulation with (**A**) binary (two-group) batch effect, (**B**) continuous batch effect, (**C**) a two-group batch effect and continuous batch effect.

To compare the performance of different methods in estimating technical artifacts, firstly we calculated the percentage of correctly estimated number of SVs. The SVA (“BE” and “Leek”) and PCA (based on different percent of variation) were considered, while in the residual RUV approach the number of factors of unwanted variation needs to be specified in advance [17]. We found that various factors impact the performance of the various methods in determining the number of SVs, such as the percentage of genes affected by batch effect, the type of batch effects (binary, continuous, both), and the sample size (**Fig 5**). The SVA (“BE”) procedure outperformed the other methods by correctly estimating the number of SVs in 70–100% of simulations. The PCA approach with SVs determined if explained > 7% of variation also behaves well in correctly estimating SVs when the percentage of genes were impacted by the batch effects was increased. The SVA method (“BE” or “Leek”) and the PCA based approach with SVs determined based on explaining >8% or >7% of variation perform similarly when the sample size and the percentage of genes affected by batch effects was large (see **Fig 5A and 5B**). Moreover, the percentage of correctly estimated SVs by SVA (“Leek”) is greater than 80% for most of the scenarios, while this percentage decreases significantly in the case when using both binary and continuous batch effects.

**Fig 6:**
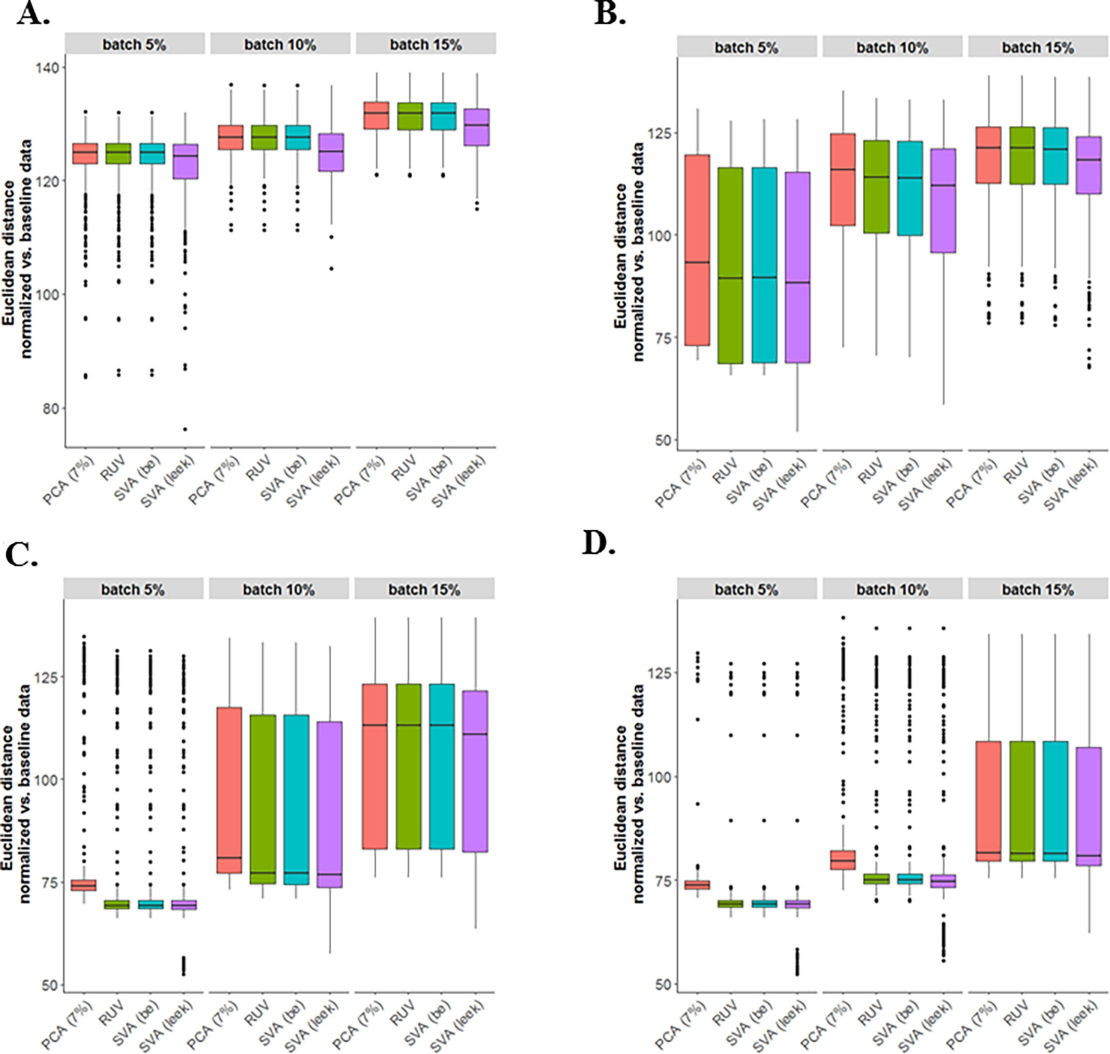
The performance of different across latent factor identification methods followed by sample normalization (SVA “BE”, SVA “Leek”, PCA-7% variation, and residual RUV) were compared. Euclidean distance between normalized and baseline data based on simulated data with both two-group and continuous batches: (**A**) N = 20, (**B**) N = 50, (**C**) N = 100, (**D**) N = 200.

After estimating the SVs, we normalized the data by fitting a linear model for log-transformed data, where the design matrix included the primary factor of interest and the estimated latent factors. The performance of different across latent factor identification methods followed by sample normalization (SVA “BE”, SVA “Leek”, PCA-7% variation, and residual RUV) were compared using the Euclidean distance as the similarity metric to measure the similarity between the normalized and baseline simulated data (i.e., data with no batch effects). To estimate the SVs based on the residual RUV method, the number of estimated SVs through the SVA (“BE”) method was used as the number of factors of technical artifacts. As presented in **Fig 6**, where both binary and continuous batch effects were used, there was more variation in the SVA (“Leek”) method compared with other methods for estimating the SVs, while the residual RUV and SVA (“BE”) methods perform similarly. The PCA (7% of variation) performed similar to the other methods, especially when the percentage of genes was affected by batch effects was large. Lastly, considering other types of batch effects (i.e., binary and continuous) may support similar results in assessing Euclidean distance between normalized and baseline data (**S4 and S5 Figs**). Note that the outliers in the boxplots of Euclidean distance demonstrate the less similarity between the batch effect normalized data and the baseline simulate data that depends on the truly estimate of number of SVs along with how the estimated SVs can capture the technical variation.

**Fig 7:**
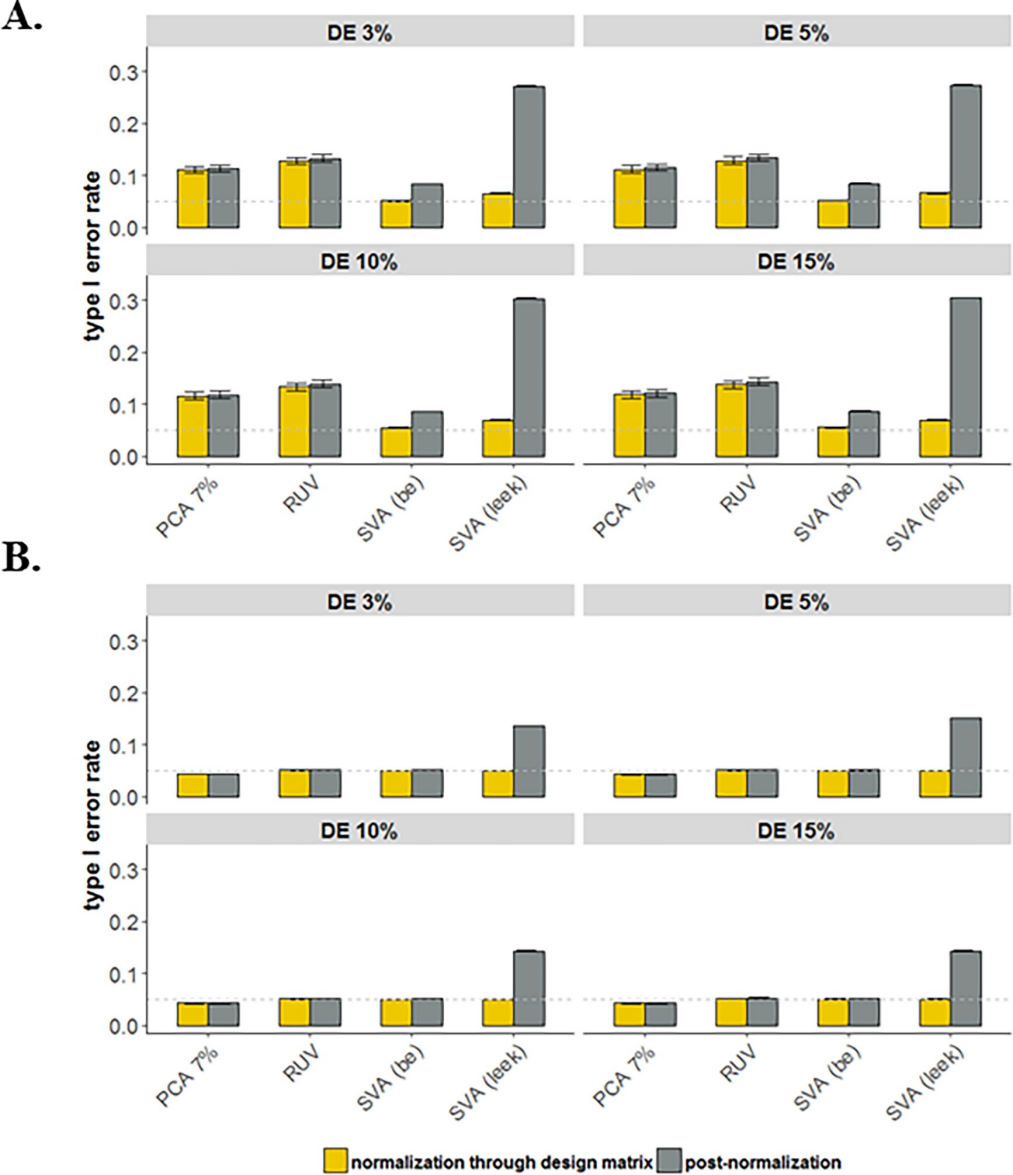
Assess the impact on association analysis the lack of correctly accounting for the loss of degrees of freedom due to across sample normalization methods, SVA (“BE” and “Leek”), residual RUV, and PCA (7% of variation); for simulated data with varying proportion of DE genes (3–15%) and taking into account both binary and continuous batches. Average empirical type I error rates over 1,000 simulated data sets: (**A**) N = 50 and (**B**) N = 200. The horizontal dashed line shows the threshold 0.05.

The last aspect of the simulation study was designed to assess the impact on association analysis the lack of correctly accounting for the loss of degrees of freedom due to normalization. For each simulated data, we considered two approaches to determine DE genes (i.e., workflow 3): (1) analysis based on post-normalization and (2) normalization for batch effects is completed through the design matrix that also includes the primary variable of interest. Note that under the workflow 3, the UQ normalization method was not considered for the simulated data. Comparison of the approaches in terms of the average of empirical type I error rates among the 1,000 simulated datasets (**S6 Fig**). **Fig 7** shows that using the batch effect normalized data increases the average type I error rate significantly (rates as high as 30%). In addition, The SVA “Leek” is the worst performing method in accounting for loss of degrees of freedom due to normalization, since for some simulated datasets, this method considerably overestimates the number of SVs compared with other methods. Moreover, changes in the proportion of DE genes do not show any noticeable differences in the type I error rate. However, as expected the impact of decreases in the average type I error rate in the case of large sample sizes (N ≥ 200).

## Discussion

Despite, some studies showing that RNA-Seq data do not need complicated normalization [2], in practice normalization has been shown to have a great influence on the analysis of gene expression data generated using RNA-Seq technology. Recently, various normalization methods have been developed, while the increasing number of such methods makes it challenging for researches to decide which normalization method to use for their data analysis. In addition, none of the previous studies did the comprehensive comparison of the library size and across sample normalization methods, where the impact of loss of degrees of freedom due to normalization for downstream differential expression analysis was taken into account. Therefore, to provide recommendations for choosing among these methods, we assessed the various methods for library size normalization (UQ, TMM, and RLE) and determining latent artifacts (SVA “BE”, SVA “Leek”, residual RUV, and PCA) in the data using both real and simulated data. Moreover, we assessed the impact of not accounting for the loss of degrees of freedom due to normalization and the impact on the type I error rate for differential expression analysis. Based on this study, we suggest that in addition to completing library size normalization, researchers assess known and unknown technical artifacts in the data, and if needed, complete across sample normalization. Moreover, we suggest that instead of using the adjusted (or normalized) data in differential analysis, that researchers include the known technical artifacts as the covariates along with the primary factors of interest in the design matrix, which correctly accounts for the loss of degrees of freedom.

In applying the library size normalization methods (UQ, TMM, and RLE) to the CESC data, we showed that these methods performed similarly (**Fig 2**). As we mentioned before, it is important to keep in mind that TMM and RLE methods rely on an assumption that most genes are equivalently expressed in the samples [21,34]. In practice, checking these assumptions is difficult and such assumptions are often not met. In addition to completing library size normalization for CESC data, we considered the effect of different across-latent-factor identification methods on the differential expression analysis, where the goal is to assess the impact of correctly accounting for loss of degrees of freedom due to normalization. We chose the same number of latent factors to be included in all three normalization methods (i.e., top three latent factors). In practice, researchers will need to determine statistically or empirically how many latent factors they wish to adjust for in their analyses. Researchers should also be aware that some of the estimated latent factors may not represent technical biases, but rather true biology represented in the data. Thus, it is important to adjust for any known biological factors of interest prior to estimation of latent factors. We found in the CESC data that the residual RUV and PCA analyses produced more similar DE results, compare to SVA (“Leek”). In addition, more DE genes were detected when normalization was completed for library size, known batch effects and unknown latent factors, compared to using the log-transformed UQ normalized data.

Finally, the simulated data results showed that various factors impact the performance of the various methods in determining the number of SVs (SVA “BE”, SVA “Leek”, PCA), such as the percentage of genes affected by batch effect, the type of batch effects, and the sample size. The SVA (“BE”) procedure outperformed the other methods by correctly estimating the number of SVs, while increases in the sample size and the percentage of genes affected by batch effects can improve the performance of PCA based approach with SVs determined based on explaining >8% or >7%. In addition, comparing the performance of SVA (“BE” and “Leek”), PCA-7% variation, and residual RUV methods followed by sample normalization using Euclidean distance showed that SVA (“Leek’) method had more variation compared with other methods. Moreover, using the normalized data (SVA “Leek”) increased considerably the average type I error rate due to overestimate the number of SVs for some simulated datasets.

Lastly, in completing the DE analysis step, if possible we suggest to incorporate the estimated unknown technical variations along with the known technical variation (batch ID) and the primary factor of interest in the design matrix to correctly reflect the loss of degrees of freedom due to the normalization. The accounting for the loss of degrees of freedom is often overlooked, resulting in researchers overestimating their statistical power. Thus, it is imperative for RNA-Seq studies to incorporate good experimental design principals to reduce the need for across sample data normalization.

## Conclusion

We recommend the assessment and, if required, the use of across sample normalization methods, in addition to the library size normalization, in the analysis of RNA-Seq data. As often one does not know all the technical factors that might impact gene expression experiments, assessment of both known technical artifacts (e.g., batch ID) and unknown latent factors is recommended. In addition, we recommend that instead of using the normalized data in differential expression analysis, that researchers include the known technical artifacts as the covariates along with the primary factors of interest in the design matrix, which correctly accounts for the loss of degrees of freedom. Lastly, the ability to complete normalization does not replace the need for the incorporation of good experimental design principals in completing RNA-Seq experiments.

## Supporting information

S1 Fig (**A**) FPKM/RPKM normalization for gene length. (**B**) Illumnia sample multiplexing overview; two representative DNA fragments from two unique samples, each attached to a specific barcode sequence that identifies the sample from which it originated (i). Libraries for each sample are pooled and sequenced in parallel. Each new read contains both the fragment sequence and its sample identifying barcode (ii). Barcode sequences are used to de-multiplex, or differentiate reads from each sample (iii). Each set of reads is aligned to the reference sequence (iv).
(TIF)

S2 Fig Plots of the top two PCs with the proportion of total variation on CESC data to cluster primary factor of interest (keratin-low and keratin-high groups) after adjusting the library size (UQ) and removing the effect of batch ID and estimated SVs by use of (**A, C**) residual RUV and (**B, D**) SVA (“Leek”) through normalization. Each point is colored based on the two keratin groups (**A, B**) and 14 levels of known batch ID (**C, D**).
(TIF)

S3 Fig Overlap DE genes (adjusted p < 0.05) found under the UQ library size normalization method and the methods for determining the latent artifacts: (**A**) PCA, (**B**) residual RUV, and (**C**) SVA (“leek”) for CESC data: (blue) the normalized data (i.e., adjusted for known batch ID and estimated latent artifacts), where design matrix contains only the primary factor of interest (i.e., keratin-low and keratin-high groups), (green) log-transformed UQ normalized data, where the design matrix contains the estimated technical artifacts and the known technical artifact (batch ID) along with the primary factor of interest.
(TIF)

S4 Fig The performance of different across latent factor identification methods followed by sample normalization (SVA “BE”, SVA “Leek”, PCA-7% variation, and residual RUV) were compared. Euclidean distance between normalized and baseline data based on simulated data with binary batch: (**A**) N = 20, (**B**) N = 50, (**C**) N = 100, and (**D**) N = 200.
(TIF)

S5 Fig The performance of different across latent factor identification methods followed by sample normalization (SVA “BE”, SVA “Leek”, PCA-7% variation, and residual RUV) were compared. Euclidean distance between normalized and baseline data based on simulated data with continuous batch: (**A**) N = 20, (**B**) N = 50, (**C**) N = 100, and (**D**) N = 200.
(TIF)

S6 Fig Assess the impact on association analysis the lack of correctly accounting for the loss of degrees of freedom due to across sample normalization methods, SVA (“BE” and “Leek”), residual RUV, and PCA (7% of variation); for simulated data with varying proportion of DE genes (3–15%) and taking into account both binary and continuous batches. The empirical type I error rates among 1,000 simulated data sets: (**A**) N = 50 and (**B**) N = 200. The horizontal dashed line shows the threshold 0.05.
(TIF)

S1 Table Simulation study parameters. (XLSX)

S1 File Simulation of gene expression data. (DOCX)
