# Supplementary figures and images for: Comparison of normalization approaches for gene expression studies completed with high-throughput sequencing
Source: PLoS One. 2018 Oct 31;13(10):e0206312. doi: 10.1371/journal.pone.0206312 (PMC6209231; doi:10.1371/journal.pone.0206312)

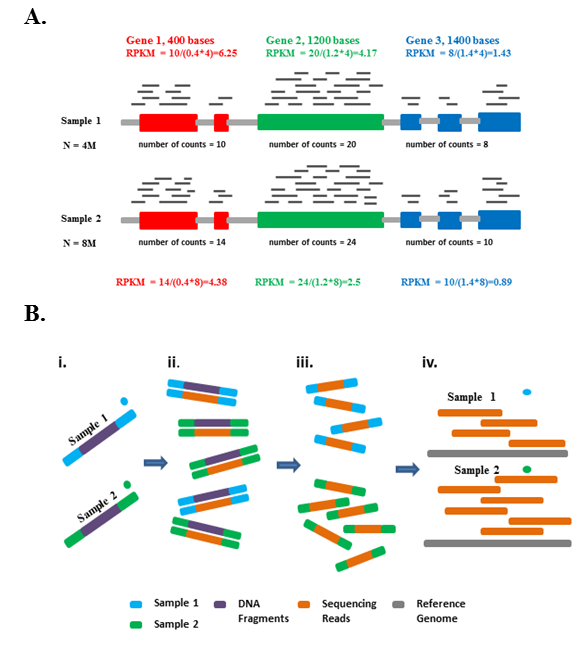

Supplement: S1 Fig — (A) FPKM/RPKM normalization for gene length. (B) Illumnia sample multiplexing overview; two representative DNA fragments from two unique samples, each attached to a specific barcode sequence that identifies the sample from which it originated (i). Libraries for each sample are pooled and sequenced in parallel. Each new read contains both the fragment sequence and its sample identifying barcode (ii). Barcode sequences are used to de-multiplex, or differentiate reads from each sample (iii). Each set of reads is aligned to the reference sequence (iv). (TIF) [file pone.0206312.s001.tif]

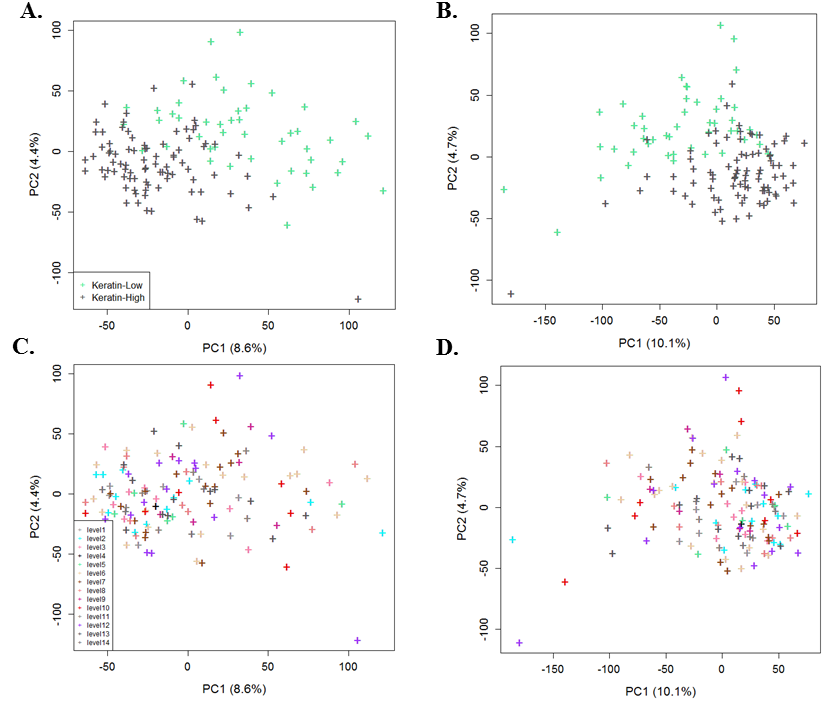

Supplement: S2 Fig — Plots of the top two PCs with the proportion of total variation on CESC data to cluster primary factor of interest (keratin-low and keratin-high groups) after adjusting the library size (UQ) and removing the effect of batch ID and estimated SVs by use of (A, C) residual RUV and (B, D) SVA (“Leek”) through normalization. Each point is colored based on the two keratin groups (A, B) and 14 levels of known batch ID (C, D). (TIF) [file pone.0206312.s002.tif]

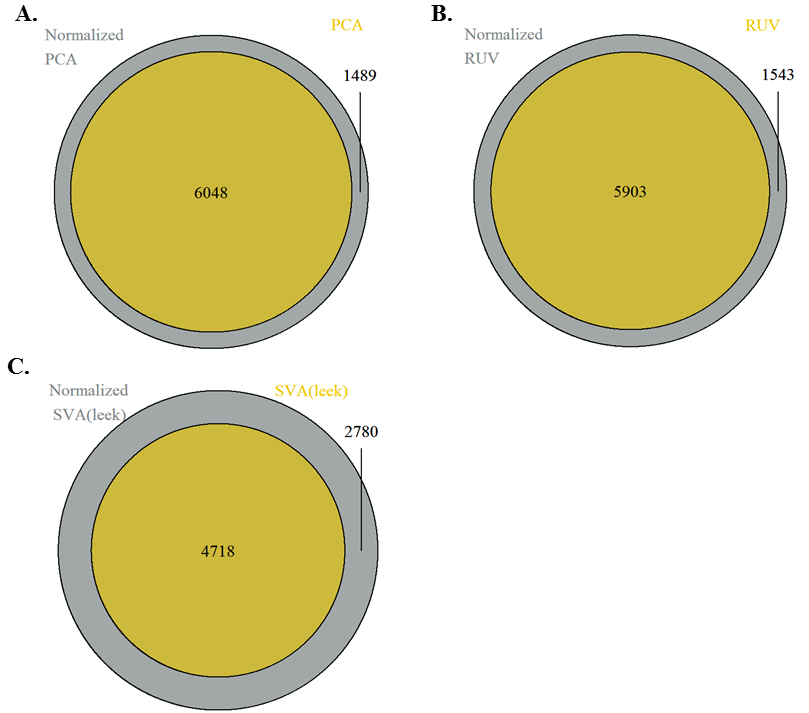

Supplement: S3 Fig — Overlap DE genes (adjusted p < 0.05) found under the UQ library size normalization method and the methods for determining the latent artifacts: (A) PCA, (B) residual RUV, and (C) SVA (“leek”) for CESC data: (blue) the normalized data (i.e., adjusted for known batch ID and estimated latent artifacts), where design matrix contains only the primary factor of interest (i.e., keratin-low and keratin-high groups), (green) log-transformed UQ normalized data, where the design matrix contains the estimated technical artifacts and the known technical artifact (batch ID) along with the primary factor of interest. (TIF) [file pone.0206312.s003.tif]

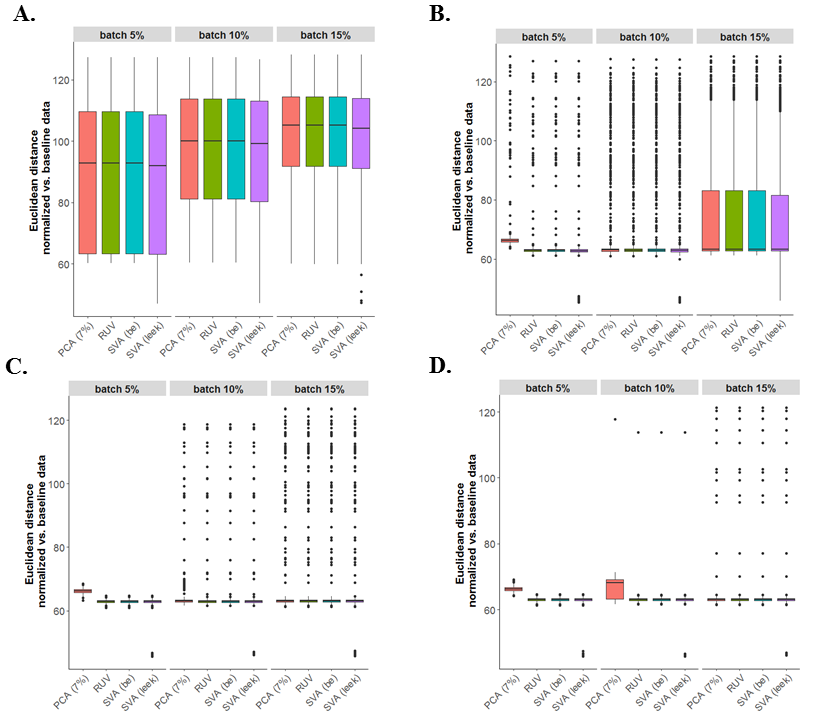

Supplement: S4 Fig — Euclidean distance between normalized and baseline data based on simulated data with binary batch: (A) N = 20, (B) N = 50, (C) N = 100, and (D) N = 200. (TIF) [file pone.0206312.s004.tif]

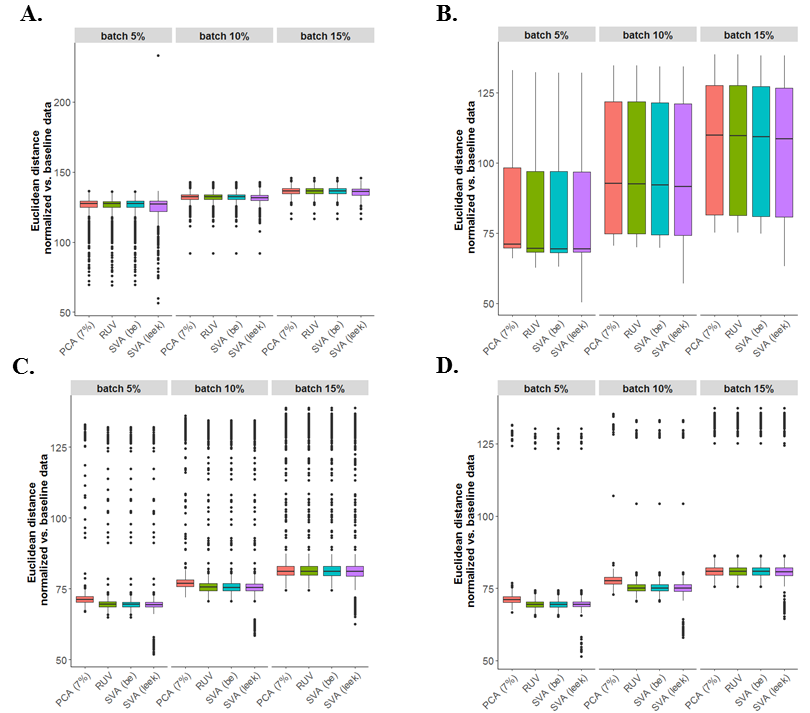

Supplement: S5 Fig — The performance of different across latent factor identification methods followed by sample normalization (SVA “BE”, SVA “Leek”, PCA-7% variation, and residual RUV) were compared. Euclidean distance between normalized and baseline data based on simulated data with continuous batch: (A) N = 20, (B) N = 50, (C) N = 100, and (D) N = 200. (TIF) [file pone.0206312.s005.tif]

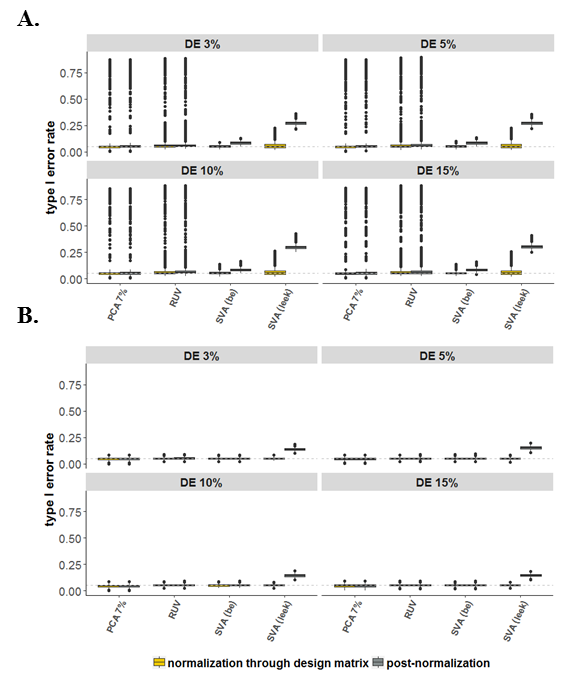

Supplement: S6 Fig — The empirical type I error rates among 1,000 simulated data sets: (A) N = 50 and (B) N = 200. The horizontal dashed line shows the threshold 0.05. (TIF) [file pone.0206312.s006.tif]
