# Supplementary material for: Comparison of normalization approaches for gene expression studies completed with high-throughput sequencing
Source: PLoS One. 2018 Oct 31;13(10):e0206312. doi: 10.1371/journal.pone.0206312 (PMC6209231; doi:10.1371/journal.pone.0206312)
Supplement: S1 File — (DOCX) [file pone.0206312.s008.docx]

**S1 File: Simulation of Gene Expression Data**

Farnoosh Abbas Aghababazadeh

27 August, 2018

Table of Contents

[1. Summary of function 1](#_Toc523148042)

[2. References 2](#_Toc523148043)

## Summary of function

We generated the simulated counts data (Leek, 2014) based on the estimated parameters using TCGA CESC data. Generate a simulated data set based on known model parameters as follows:

- *mu*: baseline mean expression for negative binomial model (using maximum likelihood method to estimate the parameter from CESC data)
- *theta*: size baseline dispersion expression for negative binomial model (using maximum likelihood method to estimate the parameter from CESC data)
- *p0*: a vector of the probabilities a count is zero
- *m*: number of genes to simulate
- *n*: number of samples to simulate
- *mod*: model matrix you would like to simulate from without an intercept
- *beta*: set of coefficients for the model matrix
- *seed*: optional seed to set (for reproducibility)

Lastly, the data matrix with counts for genes in rows and samples in columns, named *counts0* (baseline data) and *counts* (affected genes by batch and/or primary biological variables) are returned.

creat_counts <- function(mu, theta, p0, m = NULL, n = NULL, mod = NULL, beta = NULL,
 seed = NULL) {
 if (!is.null(seed)) {
 set.seed(seed)
 }
 if (is.null(m) | is.null(n)) {
 stop(.makepretty("create_read_numbers error: if you don't specify\n mod and beta, you must specify m and n.\n"))
 }
 m = dim(beta)[1]
 n = dim(mod)[1]
 index = sample(1:length(mu), size = m)
 mus = mu[index]
 p0s = p0[index]

 mumat0 = log(mus + 0.001) %*% t(rep(1, n))

 ind = !apply(mod, 2, function(x) {
 all(x == 1)
 })
 mod = cbind(mod[, ind])
 beta = cbind(beta[, ind])
 mumat = log(mus + 0.001) + beta %*% t(mod)

 sizevec = theta
 sizemat = matrix(sizevec, nrow = m)
 counts0 = matrix(NA, nrow = m, ncol = n)
 counts = matrix(NA, nrow = m, ncol = n)

 for (i in 1:m) {
 counts[i, ] = rbinom(n, prob = (1 - p0s[i]), size = 1) * rnbinom(n,
 mu = exp(mumat[i, ]), size = sizemat[i, ])
 counts0[i, ] = rbinom(n, prob = (1 - p0s[i]), size = 1) * rnbinom(n,
 mu = exp(mumat0[i, ]), size = sizemat[i, ])
 }

 return(list(counts0 = counts0, counts = counts))
}

## References

Leek, Jeffrey T. 2014. “Svaseq: Removing Batch Effects and Other Unwanted Noise from Sequencing Data.” *Nucleic Acids Research* 42 (21). Oxford University Press: e161–e161.
